# An analysis of African Swine Fever consequences on rural economies and smallholder swine producers in Haiti

**DOI:** 10.3389/fvets.2022.960344

**Published:** 2022-10-12

**Authors:** Ralph P. Jean-Pierre, Amy D. Hagerman, Karl M. Rich

**Affiliations:** ^1^Department of Agricultural Economics, Oklahoma State University, Stillwater, OK, United States; ^2^Master of International Agriculture Program, Ferguson College of Agriculture, Oklahoma State University, Stillwater, OK, United States

**Keywords:** African Swine Fever, Haiti, partial equilibrium model, animal health, economic impact analysis

## Abstract

African Swine Fever (ASF) causes high mortality and often results in strict culling policies for affected pigs and international market restrictions. It took more than 25 years for swine inventories in Haiti and the Dominican Republic to recover from an ASF outbreak that took place from 1978 to 1984. The 2021 outbreaks in the Dominican Republic and Haiti pose threats to animal health, livestock markets, and producer livelihoods. A partial equilibrium Haitian pig sector model (HPM-2021) was developed to assess the economic impacts of a 2021 Haitian ASF outbreak of a similar size to the 1980s outbreak. The dynamic model examines ASF impacts from 2021 to 2024, through 100 iterations of stochastic supply shocks, and three specific demand shocks. Recovery alternatives are assessed through 2030, and outbreaks and recovery outcomes are compared to a baseline reflecting 2019 trends. The analysis includes economic effects on national pork and maize in Haiti, the Dominican Republic, the rest of the Caribbean, and the rest of the world. Findings demonstrate higher vulnerabilities of the traditional sector to ASF-related disruptions. The inflated prices generated by pork production shortfalls are an opportunity to accelerate income growth for remaining traditional pig producers. When there is no consumer avoidance, the production losses caused by ASF generate high prices and contribute to a minimum of 49% increase in traditional sector revenue, and a minimum of 2.22% revenue growth in the commercial sector from the 2019 base year. Nevertheless, the potential for consumer avoidance of pork cause prices to decrease and offset those gains by as much as 90% in the traditional sector and 44% in the commercial sector. Smaller commercial sector impacts derive from different elasticities. ASF-induced high prices also lead to increased consumer expenditure losses by up to 200% over the outbreak period. Nevertheless, consumer expenditures tend to recover instantaneously with ASF eradication. Due to persisting demand shocks, producers will earn up to 0.3% lower than baseline levels income from 2027 to 2030. Few models evaluate the economic impacts of health response policies in less developed countries like Haiti. HPM-2021 results highlight ASF impacts on prices, which can benefit certain producers and disincentivize on-farm disease reporting. Slow recovery and consumer avoidance of pork are detrimental to long-term swine industry survival, producer livelihoods, and the overall rural economy.

## Introduction

In 2021, nearly four decades after the 1980s high-cost African Swine Fever (ASF) eradication, the USDA Foreign Animal Disease Diagnostic Laboratory confirmed new ASF cases on the Hispaniola Island (1). Outbreaks were confirmed in the Dominican Republic in July by the USDA Foreign Animal Laboratory from samples collected in May (2). The first infections were confirmed in the northeastern provinces, near the Haitian border. This proximity raised concerns about the risks of swine infection in Haiti, where swine production remains a crucial economic sector. In September, the Chief Veterinary Officer in Haiti also confirmed ASF cases in a province bordering the Dominican Republic. By March 2022, the virus had been detected in 29 Dominican provinces and 8 of 10 geographical departments of Haiti (3). According to a report in Ohio's Country Journal, the World Organization for Animal Health reported that the detected ASF-infected pigs had been immediately slaughtered, and zoning had been applied, with surveillance of the containment zone (1, 4).

The last ASF outbreak on Hispaniola Island was in 1978-1984. Haitian smallholder swine farmers experienced long-lasting hardships due to the lack of diversification in their income and asset portfolios (5). Before the first ASF outbreaks, indigenous “creole” pigs were the most plentiful farm animals. Over 80% of Haiti's population lived in rural areas, and 95% of them were smallholder swine producers (6). For most owners, pigs were an important source of wealth and cash income. Although creole pigs were relatively small—usually not exceeding 150 pounds—they constituted the backbone of the rural Haitian economy. Rural areas highly benefited from their low production costs (5). Four decades after a costly ASF eradication, the Haitian swine sector still has not completely recovered production levels from the 1978-1984 ASF outbreaks (7).

Much has changed in the global swine market and policies for disease eradication, yet the Haitian swine industry structure is still very similar to the 1970s (8). In rural areas, the traditional production system is dominant, with low biosecurity and disease prevention measures. Pigs continue to play a major role in reducing food insecurity, maintaining soil fertility, and empowering social equality. Before the 2021 ASF discovery, Haiti already faced challenges of food insecurity, unemployment, and high vulnerability to social and natural hazards. Economic and social development continued to be hindered by political instability (9). Consequently, the country has one of the lowest Human Development Index scores (0.51) and GDP per capita ($1,149 per year). According to the World Bank, the COVID-19 pandemic further deepened the economic contractions reported in the previous years (10). The recent socio-economic challenges emphasize the need to apply efficient ASF control measures in the current outbreak that preserve swine producer livelihoods. In 2019, pigs have contributed up to 11% of the total domestic livestock output (11). Historically, as much as 70% of Haiti's rural population raised pigs, often in small numbers per farm most commonly raised for wealth creation and investment against future expenditures (8).

Many owners rely on swine production for their animal-derived nutrients (5). Besides being a high-protein food, pork is also a good source of potassium, zinc, phosphorus, and vitamin B6, which are crucial elements for fighting chronic nutrient deficiencies (12). Despite the importance of the swine industry in the overall economy, smallholder producers often lived in rural communities lacking consistent access to essential services. In 2019, rural areas received only 15% of the country's electrical power, 16% of the modern health facilities, and 20% of the education-related expenditures (10, 13).

Although there is a need to assess the impact of ASF discovery today, as well as identify suitable eradication policies, there are no economic models of the Haitian pig sector's response to ASF shocks. The purpose of this research is to assess the economic consequences of the 2021 ASF outbreaks on swine production and consumption, and the cost-effectiveness of prospective control measures in Haiti. The Haitian pig sector partial equilibrium model (HPM-2021) was developed to simulate the dynamic effects of ASF and adopted control measures. The research consists of a baseline (no-outbreak) scenario that projects the 2019-2020 changes in production, consumption, and prices of traditional pigs, commercial pigs, and maize through 2030. The economic impacts of ASF are estimated through numerous alternative scenarios based on Haiti's previous experiences with ASF, and policies applied by other affected countries in the past.

The paper starts by outlining ASF's first introduction in Haiti in 1978 and the creation of the Projet pour l'Eradication de la Peste Porcine Africaine et pour le Développement de l'Elevage Porcin (PEPPADEP) in 1981. The PEPPADEP was launched by the Government of Haiti, in collaboration with the governments of Canada, Mexico, and the United States, the Food and Agriculture Organization of the United Nations, the Inter-American Institute for Cooperation on Agriculture, and the Interamerican Development Bank. The analysis of the 1980s outbreaks in Haiti and international ASF eradication experiences lay out the conditions to simulate the impacts of ASF-related shocks in HPM-2021. The quantitative results describe the potential effects on livelihoods from the 2021 ASF outbreaks. The outcomes may enhance the development of measures that could cost-effectively decrease ASF consequences to producers. Finally, the study includes an analytical framework that examines alternative policies, not pursued by the PEPPADEP, to reconstitute the Haitian swine industry after the 2021 outbreak.

## Background of ASF infection

ASF had been reported for the first time in Africa in the 1900s and existed for many years in the wild indigenous pig (warthogs) population and the soft tick *Ornithodros moubata porcinus* (14, 15). Two major incursions of the disease outside of the African continent occurred in 1960 and 2007 (16, 17). From this second incursion, ASF was diagnosed in multiple European countries and China, the world's largest pork producer and consumer, triggering infections in other Asian countries (18). During both events, the island of Hispaniola experienced the introduction of ASF into Haiti and the Dominican Republic (19, 20).

Among Transboundary Animal Diseases affecting swine, ASF is one of the most detrimental for infected livestock populations, with the potential for high socioeconomic consequences (21, 22). A commercial vaccine is yet to be available for ASF prevention (23). However, recent studies indicate that current vaccine candidates optimistically show efficacy in protecting animals against the homologous virulent strains of ASF (24).

Past ASF experiences demonstrate that the control policies have greatly influenced eradication timeliness and economic costs to the affected countries (25). A contiguous culling strategy was not uncommon in the 20th century and early in the 21st century for ASF and other highly contagious diseases. Contiguous culling involves the depopulation of infected premises and surrounding premises. This culling approach was employed in Hispaniola Island and ensured eradication in a span of 3 years. Contiguous culling was resource intensive, and compensation was paid to farmers for animals slaughtered (25). The 1980s Haitian ASF eradication cost $17 million in 4 years (5). The implementation of an efficient repopulation strategy was crucial for the swine industry's long-term survival under such an intensive depopulation strategy to prevent the permanent loss of livelihoods. Much can be learned from examining prior responses to ASF in Haiti, and lessons learned from other countries.

The 1970s represent a historical peak in Haitian pig inventories (26). Dominican government officials detected ASF in pig herds in 1978. As reported by USAID, ASF was introduced by means of infected meat transported by plane from Mediterranean countries. Although the authorities rapidly opted for the extermination of the pig population, ASF detection in the Dominican Republic raised serious concerns for the Republic of Haiti. In the Fall of 1978, the government of Haiti confirmed cases of ASF on the border of Haiti and the Dominican Republic (5).

Villeda et al. (27) compare the hemostatic defects leading to hemorrhage in the ASFV strain that infected the Dominican Republic and Haiti in 1978, with a strain of different virulence that spread in Malta during the same period. Investigations confirmed that the mortality rates and severity of clinical observations were higher in infected Dominican pigs. In the light of the risks that faced swine producers, the Haitian Ministry of Agriculture and the Military decided to enforce containment measures. The Ministry ordered the extermination of all pigs located within 15 km from the border in an aggressive contiguous culling strategy (6). The New York Times reported that more than 28,000 pigs (2% of total production) had been slaughtered between 1978 and 1981 in a systematic effort to contain ASF (28). No compensation had been allocated to farmers in this first round of depopulation. Due to that lack of incentives to report on-farm disease, along with limited preparedness, biosafety protocols, and educational efforts, ASF extended throughout the country's nine geographical departments by 1981, causing a crisis in the entire swine sector (5).

In addition to ASF prevention challenges in Haiti, the virus threatened to spread to other countries. In particular, the disease threatened Haiti's northern neighbors, most notably the United States and Canada. These countries were expanding commercial swine industries producing pork and pork products for export markets at the time and had much to lose should the virus enter their borders and potentially impact their multi-billion-dollar swine industries. Ebert (29) estimated that the United States could face up to $5 billion worth of damages should ASF reach the country. Based on this reality, the governments of Canada, Mexico, and the United States, collaboratively with the American Institute for Cooperation on Agriculture, offered immediate financial support to the Haitian government for the elaboration of an effective ASF eradication plan. The PEPPADEP was initiated in 1981 (5).

The GOH launched PEPPADEP with the contribution of North American neighbors, through multiple international organizations. The project consisted of two phases. The first aim was to eradicate ASF by 1984 through the extermination of indigenous pigs (5). The second phase was to reconstitute the swine industry and introduce higher productivity swine breeds. International organizations agreed that smallholder farmers in Haiti would not be able to count on pigs as a sustainable livelihood unless ASF was eradicated from the country (5). Additionally, from the international agricultural community's standpoint, ASF eradication in Haiti was an exceptional occasion to improve the national productivity of swine. Before ASF was detected in Haiti, the pig population was estimated at 1.6 million, mostly owned by smallholder farmers living in rural areas (26).

At the completion of the program, a total of 384,391 pigs had been slaughtered for $9,548,860 in compensation paid (Table 1). An estimated 600,000 additional pigs died from ASF infection (29). The PEPPADEP completed the first phase of its mission in 1984. Through restrictive pig movement and massive depopulation, ASF was officially eradicated from the island in 1984 (5, 19).

**Table 1:** Summary of pigs (a) killed and compensation paid during slaughter from 1981 to 1984 as part of the PEPPADEP campaign in Haiti.

	**Pigs slaughtered in head**	**Compensation paid (US$)**
Adults	168,007	$6,720,280
Young	116,444	$2,328,880
Piglets	99,940	$499,700
Total	384,391	$9,548,860

^a^In 1978, prior to ASF, the total swine inventory in Haiti was 1.6 million total head. Source: The data in this table derive from the USAID Haiti “Interim Swine Repopulation” report, 1983 (5).

Following ASF eradication, a repopulation program was launched to alleviate smallholder producers who had been economically devastated by the indigenous pigs' extermination and left with limited sources of breeding stock. The second phase of PEPPADEP's mission was to import, multiply, and distribute specific-pathogen-free hogs to farmers. Pure lines of Duroc, Yorkshire, Hampshire, and Berkshire were imported from Noth America. The USAID anticipated many challenges to the repopulation plans:

- The lack of swine holding facilities to be used as secondary multiplication centers.
- The limited well-trained personnel to carry out the project.
- The adaptation of exotic pigs to farmers' poor nutrition methods after distribution (5).

**Figure 1:**
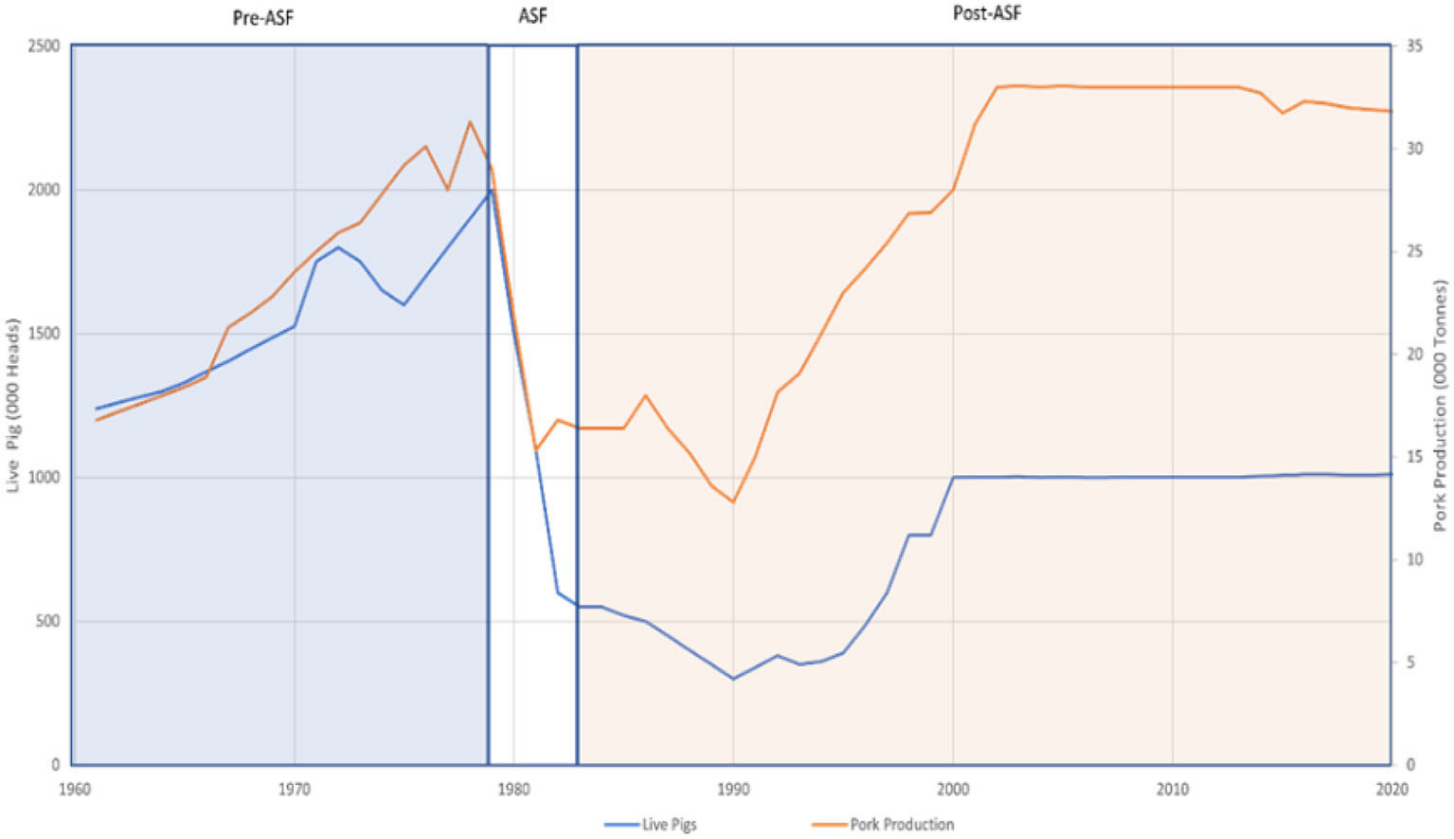
Comparison of live pigs and pork processed before, during, and after African Swine Fever in Haiti.

Various critics argued that the program failed to redevelop the Haitian swine industry (6, 29). Post-ASF data indicate that the country failed to reach pre-ASF live pig volumes four decades after eradication (Figure 1). On the other hand, owing to new breeds' larger size and higher meat productivity, domestic pork supply (in tons) surpassed pre-ASF levels in 2002 and remained stable in the following periods.

Figure 1 compares the level of yearly meat processed and live pigs in Haiti from the period preceding the 1978 ASF introduction to the decades following the 1984 eradication. Processed pork are all products deriving from slaughtered pigs. In 1978 and 1979, Haitian processed pork and live pig both reached record volumes. ASF-related losses caused a rapid decline in the pig population and meat shortfalls throughout the country. In the 6 years leading to eradication, live pigs and meat processed dropped by 71% and 43%, respectively. Imported pork helped offset effects for consumers, but the impacts for producers were significant.

The post-ASF period data demonstrate the difficulties to reconstitute the swine industry. The massive loss of Haiti's indigenous breed has had detrimental socio-economic impacts on smallholder producer livelihoods as evidenced by indicators such as nutrition security, income, and social equality (5). The PEPPADEP's swine repopulation initiatives began in 1985, with the distribution of improved breeds. Inadequate feeding and veterinary practices on Haitian farms adversely impacted the new breeds' well-being. Some of the breeds were not suited for yearly high temperatures. Also, certain Haitian cultural celebrations and voodoo ceremonies required totally black pigs. This served as a motive for farmers' refusal of white Yorkshires, white striped Hampshires, and red Durocs (5).

These impediments particularly hindered the PEPPADEP's swine repopulation efforts. Consequently, live pigs and meat processed continued to decrease until 1990, and the post-ASF averages remained lower than the previous periods. In addition to a direct effect on meat availability, the post-ASF period was marked by worsening socioeconomic conditions in rural areas. ASF-related income losses caused a 14% drop in primary school enrollment, and food insecurity reached 47% (30). The drop in living standards induced an acceleration of rural out-migration. Two decades after the discovery of ASF, the rural share of the country's total population declined by 15% (31). Haiti's experience with ASF suggests that smallholder swine farmers, hit by the outbreaks and eradication procedures, struggled to recover. The new (2021-2022) outbreak of ASF also threatens the Haitian swine industry. This study uses the lessons learned from historical outbreaks and a new modeling framework to examine the potential impacts of the 2021-2022 outbreak.

## Methods and data

### HPM description

The Haitian Pig Model (HPM-2021) is an adaptation of the International Livestock Research Institute's Vietnam pig model that is used to quantify the dynamic effects of ASF infection and control policies at the sector level (32). It is a three-sector, four-region, partial equilibrium model that investigates the dynamics of traditional pigs, commercial pigs, and their interactions with maize, from the supply and demand perspective. Smallholder pigs, often raised for meat and sold in rural traditional wet markets, are classified in the “traditional pig” sector. This sector accounts for the majority of pigs supplied in Haiti and is a substantial source of employment for rural populations. Pigs raised in larger farms, often located in urban and suburban areas, by commercially oriented producers are categorized as the “commercial pig” sector.

Various studies, in the past, have used multimarket or multicommodity models to assess the market effects of negative disease shocks on the supply of livestock within a country or region (32–34). Multimarket partial equilibrium models may also serve to capture animal diseases' shocks and mitigation effects on related sectors, such as feed. HPM-2021 investigates the direct impacts of ASF on swine populations and indirect impacts of ASF shocks on maize, used for both human and animal consumption. The study examines the impacts of ASF to supply and demand in Haiti and the Dominican Republic, through their trade interactions with the rest of the Caribbean and the rest of the world.

HPM-2021 is developed within the General Algebraic Modeling System (GAMS) framework (35). The model is used to simulate changes in the swine industry from 2019 to 2030. The base year of 2019 is selected to reduce noise in the calculated parameters due to COVID-19 impacts on the international economy. ASF spread and mitigation are assessed in five different demand and supply scenarios. Those shocks are distributed across the 2021-2024 period. The following sections describe the theoretical model, data, and scenarios.

HPM-2021 uses the mixed complementarity problem (MCP) applied in GAMS (35). A set of inequalities are formulated to exploit the complementarity links between various equations. For instance, MCP facilitates modeling the links between the domestic pork demand equation and the domestic pork supply equation under normal market conditions. MCP accommodates economic research for markets, allowing the equilibrium model as systems of non–linear equations or inequalities, which are difficult to formulate in an optimization context. Our model employs a double-log specification for the supply and demand functions (32). Because HPM-2021 estimations are based on different own and cross-price elasticity coefficients, the double-log functional form is convenient to explain the potential impacts of an expected percent change in a predictor to a response variable.

The core equations consist of maize, traditional pig and commercial pigs supply, and food demand functions. Maize is used for both human and animal consumption, including domestically produced and imported maize. The maize function includes a yield and acres planted intercept. In the model, Haitian maize production is conditioned by the acres planted, yields, own price elasticities, and cross elasticities with respect to pig prices. Traditional pig production makes up the majority of swine farms and inventory in Haiti. The livestock supply functions follow a very similar procedure to maize. They include a double-log intercept determining livestock production at base prices. Traditional and commercial pig production are related to swine supply elasticity with respect to own and cross swine output prices, and the elasticity of swine supply to the price of maize. Elasticities are critical in defining the different variations in production from price changes within and across markets. Food and feed demand functions are built similarly, using consumption data and elasticities to define dynamic changes within sectors. The core equations can be found in Supplementary material.

For scenario-building purposes, the intercepts of crop area (α), livestock supply (δ) and crop and livestock demand (ϑ) functions are transformed to emphasize the impacts of ASF shocks. HPM-2021 examines an ASF outbreak only in Haiti. *G* is the ASF-induced supply shock at time *t*_1_∈(2021,…,2024) which are the ASF outbreak years in the model. *G* is implemented in crop area intercept, α, to simulate indirect shocks of ASF to total maize supply (*J*) in Haiti (equation 1). *J* includes maize used for both animal consumption and human consumption.


(1) $$\begin{array}{l} \alpha_{jt_{1}}^{shock}=A\alpha_{jt}+\log\left(1-G_{jt_{1}}\right) \end{array}$$

*G* incorporates ASF-induced supply shocks to livestock sectors at time *t*_1_ by adjusting the intercept of livestock supply in Haiti (equation 2).


(2) $$\begin{array}{l} \delta_{lt_{1}}^{shock}=\delta_{lt}+\log\left(1-G_{lt_{1}}\right) \end{array}$$

*B*, on the other hand, is the imposed demand shock at time *t*_1_ to both maize and livestock sectors (*l*) in Haiti (equation 3).


(3) $$\begin{array}{l} \vartheta_{jlt_{1}}^{shock}=\vartheta_{jlt}+\log\left(1-B_{jlt_{1}}\right) \end{array}$$

Finally, the model resets supply shocks for each scenario draw to restrain shocks building upon each other. Shock values are detailed in **Table 3**.

### Data

The model is populated by parameters on supply and demand relationships. Income, population, nominal exchange rate, technology, and international price growth also influence variations in the model. HPM-2021 development and analysis are based on secondary data collections from numerous sources. The study uses 2019 swine and maize production oriented to rural and urban consumers collected by the FAO, through its statistical database (36). The full set of tables containing model parameters and data sources are in Supplementary material document.

Per capita food consumption is estimated based on production, trade, and commodity conversion ratios from producer weight to consumer weight gathered from FAOSTAT. Dominican producer prices (in DR Pesos) were forecasted from 2015 estimates using FAO's yearly inflation index. Prices (at any level) are not regularly collected for Haiti but are available for the Dominican Republic. The 1983 USAID “Interim Swine Repopulation” is a rare report that contains 1978-1982 Haitian swine prices. Owing to limited data availability, Haitian commodity prices are an average of the 1982 prices, adjusted for inflation and the accounted historical gap with Dominican prices. International trade prices are collected from the USDA and the Haitian Customs Office. Finally, data about socioeconomic factors that may influence market changes such as 2019 income, population growth, and nominal exchange rate derived from the World Bank database. The collected data served in further parameters' estimation, such as internal and external marketing margins, processing costs, and producer prices. Fluctuations in consumer income and output prices affect the quantity demanded and supplied in the different sectors.

Market responses to demand and supply relationships are structured through pre-calculated elasticities that quantify impact estimates over time. The systemic responses to changes, in the model, are driven by demand and supply elasticities. There is not a set of elasticities readily available for Haiti. Further limitations in data and extreme events affecting the Haitian economy create challenges in the elasticity estimation process. As a result, stochastic elasticities were generated based on 1990-2015 swine and maize market data using the Simetar simulation modeling tool (37, 38).

### Scenarios

Recovery and repopulation initiatives are simulated, from 2025 to 2030, through two specific scenarios: a restricted and unrestricted recovery scenario. The process of shaping those scenarios is detailed in the following sections.

The Described Model Consists of a Baseline That Projects 2019-2020 Trends, Driven by Changes in Population, Income, Nominal Exchange Rate, and Technology Growth Through 2030 That Are Predicted in the Absence of ASF. These Elements Influence Variations in Supply and Demand, and act on Market Prices. The Baseline Reflects a Yearly 2.48% and 2% Population and Nominal Exchange Rate Depreciation, Respectively. The Rise in Maize Production Will Prospectively Influence own Prices and pig Producers' Demand for Feeds. Forward Projections in the Baseline Are Also Guided by a Yearly per Capita Income Reduction of 3.05% Assumption (Table 2). Pre-Calculated Elasticities Are Unchanged While Variations in the Parameters Are Expected to Shift Traditional pig, Commercial pig, and Maize Production and Consumption.

**Table 2:** Factor growth assumptions from the baseline.

**Parameters**	**Baseline**
Per capita income growth	−3.05%
Population growth	2.48%
Nominal exchange rate growth	2%
Maize technology growth	0.50%
Traditional pig technology growth	0%
Commercial pig technology growth	0%
Maize world price growth	2.08%
Traditional pig world price growth	−1.32%
Commercial pig world price growth	−1.32%
Income elasticity multiplier for maize	1
Income elasticity multiplier for T. Pig	1
Income elasticity multiplier for C. Pig	1
Crop supply at time T	1
Livestock supply at time T	1
Feed demand at time T	1
Food demand at time T	1
Import at time T	1

Source: Authors' Assumptions based on historical data.

The baseline is compared to several ASF-induced linear reductions in pig supply and demand. Production and consumption shocks are imposed from 2021 to 2024 to reflect ASF outbreak losses. The model includes five alternative scenarios: an average 3% demand-only shock, a stochastic supply-only shock, and three combinations of supply and demand shocks to traditional and commercial pigs as described in Table 3.

**Table 3:** Shock description for ASF prospective impacts.

**Scenario name**	**Description of the scenario**	**Supply shock: ASF**	**Demand shock: ASF**	Supply shock: Recovery^*^	**Demand shock:** recovery^*^
Demand-0s	Average consumer avoidance from a single farm outbreak	No	3%	Unrestricted	Unrestricted
Supply-0d	Stochastic supply shocks (100 draws only)	Mean: 15% S.D: 0.03	No	Unrestricted	Unrestricted
Supply_1d	Combined stochastic supply shocks with 1% demand shock	Mean: 15% S.D: 0.03	1%	Unrestricted	Unrestricted
Supply_3d	Combined stochastic supply shocks with 3% demand shock	Mean: 15% S.D: 0.03	3%	Restricted	Restricted
Supply_6d	Combined stochastic supply shocks with 6% demand shock	Mean: 15% S.D: 0.03	6%	Unrestricted	Unrestricted

* For additional information about the restricted and unrestricted shocks, see Table 4.

The ASF supply shock selection assumes an ASF outbreak similar in size to the previous 1980s infection. Previous ASF outbreaks have posed adverse impacts on the Haitian pork supply. The historical data indicate that the supply of live pigs in Haiti dropped by an average of 15% every year from 1981 to 1984. The 100 iterations of stochastic supply shocks are distributed around an average value of 15% and a standard deviation of 0.03.

ASF does not pose a threat to human health (39). However, the presence of ASF has caused concerns about disease transmission to humans, resulting in demand shocks in other countries. The potential for consumer avoidance is considered. The stochastic ASF supply shocks are combined with a small, an average, and a high demand reduction of 1%, 3%, and 6%, respectively, to illustrate aggregate ASF effects on the swine industry (Table 3). Given the limited availability of pork consumption data in Haiti and the Dominican Republic, we calculated the pork demand variations using data from three other developing countries previously impacted by ASF: Ethiopia, Nigeria, and the Philippines. Across the selected countries, yearly pork consumption declined by 3% on average after the first outbreaks (40). The highest decrease in average pork consumption (6%) was noted in Ethiopia following the 2011 ASF detection while Nigerian pork demand was the lowest drop, 1% on average.

The timeliness of swine industry recovery from ASF often varies with the eradication strategy, reinfection risk, restocking costs, and new breeds' efficient integration. The latter particularly impeded Haiti's swine repopulation process after 1984's eradication. We developed two recovery possibilities to assess the prospective impacts of repopulation measures on the traditional sector. The recovery scopes complement the combined supply and 3% demand shock from 2025 to 2030 to reflect post-eradication production and consumption trends (Table 3).

The first of the two recovery scenarios examined what would happen if the market was allowed to freely determine the speed of recovery without barriers. The unrestricted recovery allows the model to repopulate swine production toward baseline trends in 2025 with no barriers. This scenario would necessitate immediate funding accessibility, successful breeds' adaptation to local conditions, and biosecurity measures that prevent ASF reintroduction. Alternatively, the restricted recovery scenario reflects PEPPADEP's repopulation outcome following 1984's ASF eradication. This scenario includes setbacks and lingering shocks to domestic pig production until 2030. The following Table 4 details the shock reduction extent under each recovery scenario.

**Table 4:** ASF Recovery scenarios^∧^.

**Shocks/Year**	**Unrestricted**	**Restricted**
2025	1	0.93
2026	1	0.94
2027	1	0.94
2028	1	0.95
2029	1	0.95
2030	1	0.95

∧ Each shock specifies how much pig supply differ from baseline values.

## Results

### Baseline market fluctuations in swine sectors

**Figure 2:**
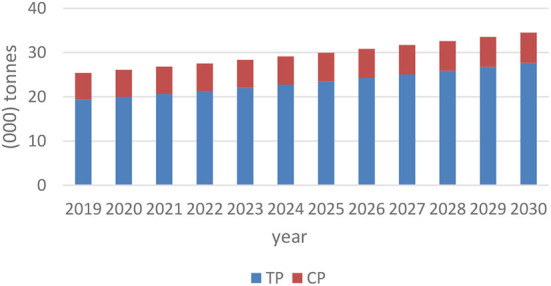
Haitian pork supply in baseline (a) TP is acronym for traditional pig and CP is an acronym for commercial pig.

HPM-2021 simulation results in the baseline show an expanding swine industry in the absence of ASF. The domestic supply of traditional and commercial pigs would be projected to increase by an average yearly rate of 3.5% (Figure 2). This is driven by an increased food demand originating from a sharp rise in the Haitian population. Despite a shrinking rural population share, due to chronic out-migration, traditional pig production still dominates up to 95% of all domestic swine inventories. Demand for pork tends to grow at the same rate as supply, 3.6% yearly (Figure 2). The pig sector in Haiti involves few trading activities with other countries, and most traditional and commercial pig supply is destined for local consumption. Consequently, HPM-2021 simulations always show an equilibrium in pork products demanded and supplied.

HPM-2021 results suggest that prices will rise progressively until 2030 in both pig sectors under baseline, non-ASF conditions. During the eleven-year period, commercial pig prices would have increased by an average of 4% per year. A faster average growth, 16% yearly, is noted in the traditional pig sector, stretching the sectoral price gap. By 2030, traditional pig price inflation will be 23 times higher than commercial pigs.

### Traditional and commercial pig price changes in alternative scenarios

**Figure 3:**
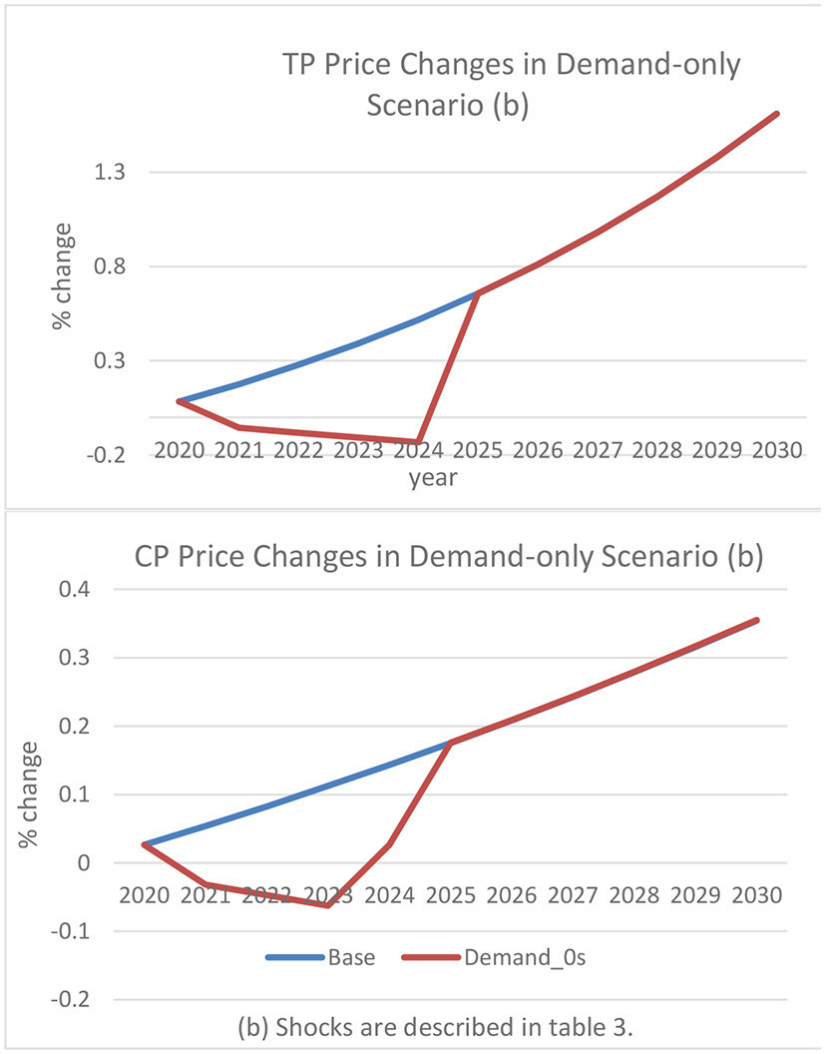
Traditional and commercial pig price changes in demand-only scenario. (b) Shocks are described in Table 3.

In the first alternative scenario, the baseline results are compared to a 3% (average) demand shock arising from a drop in consumer confidence due to ASF outbreaks. With the 3% demand shock imposed on traditional and commercial pigs, national supply decreases by an average of 14% for traditional pigs and 7% for the commercial pig sector between 2021 and 2024. The reduction in pork consumer spending triggers a substantial price decrease in both pig sectors (Figure 3). Throughout the ASF outbreak period, traditional pig prices fall by 32% compared to a 14% decrease in the commercial sector. As examined in the baseline, a demand shock with no accounted supply disruptions from ASF is expected to affect the traditional pig sector more than the commercial pig sector.

The demand shock to the pig industry, albeit relatively small, can play a sizeable role in limiting the price effects noted in the baseline. The reduction in supply resulting from lower demand in both sectors leads to prices falling by nearly one-third in the traditional sector. Sharper losses in the commercial sector allow recovery to begin earlier, in 2023, despite demand shocks persisting until 2024 (Figure 3). The unrestricted recovery scenario indicates an immediate price upturn from the demand shocks in 2025. The prompt recovery would highly limit the revenue losses, notably in the smallholding sector. Funding constraints and cultural adversities in Haitian swine production, however, minimize the likelihood of an immediate recovery.

**Figure 4:**
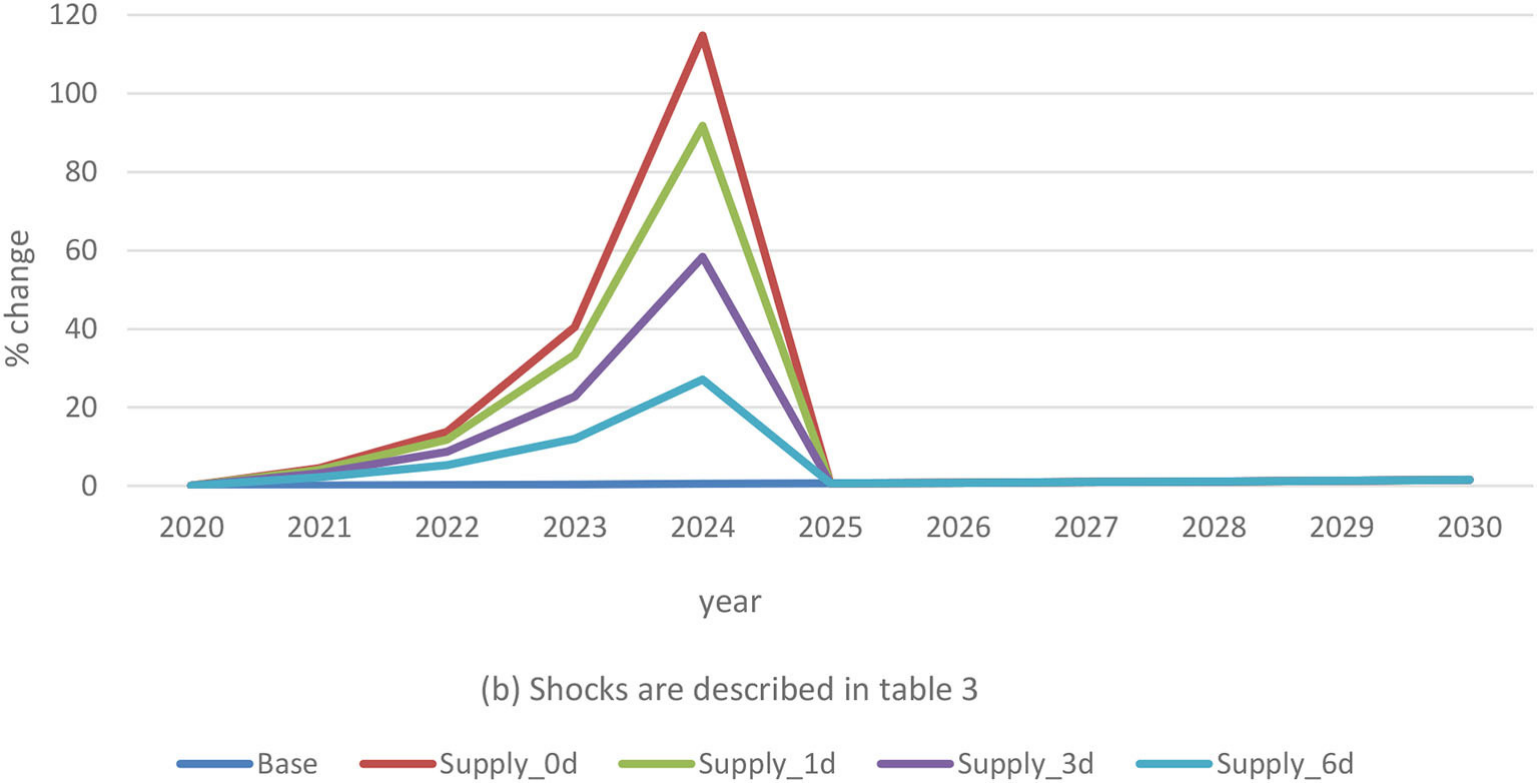
Traditional pig price changes in combined scenarios. (b) Shocks are described in Table 3.

High mortality and massive swine depopulation associated with ASF portend shocks to pork supply that influence economic impacts on farmers. The results show that a 15% traditional pig supply-only reduction lifts prices by as much as 118% compared to baseline levels (Figure 4). Adjustment with the three demand shocks limits the supply-induced price inflation by various magnitudes. A 1% consumer avoidance contributes to lowering the supply-only shock effect by an average of 24%. The 3% average demand shock offsets the supply-related price effect by 59% during the outbreak period. Finally, when combined with a 6% demand reduction, the ASF supply impact on price decreases by an average of 90%.

The supply-driven high prices during ASF outbreaks in the traditional pig sector have opposite implications for Haitian producers and consumers of pork products. For smallholder producers, the increased prices are an opportunity to collect further revenues. As the results suggest, the possibility for higher producer revenues may increase with consumer avoidance. On the other hand, more expensive food negatively affects consumers' spending ability. The demand-only scenario shows a decreasing trend in the per capita income. Rising prices generated by the ASF supply shocks to traditional pigs may contribute to ongoing poverty in rural communities. The disparity in price variation to shocks in the post-ASF period is associated with new pig production offtake under challenging feeding and veterinary conditions.

**Figure 5:**
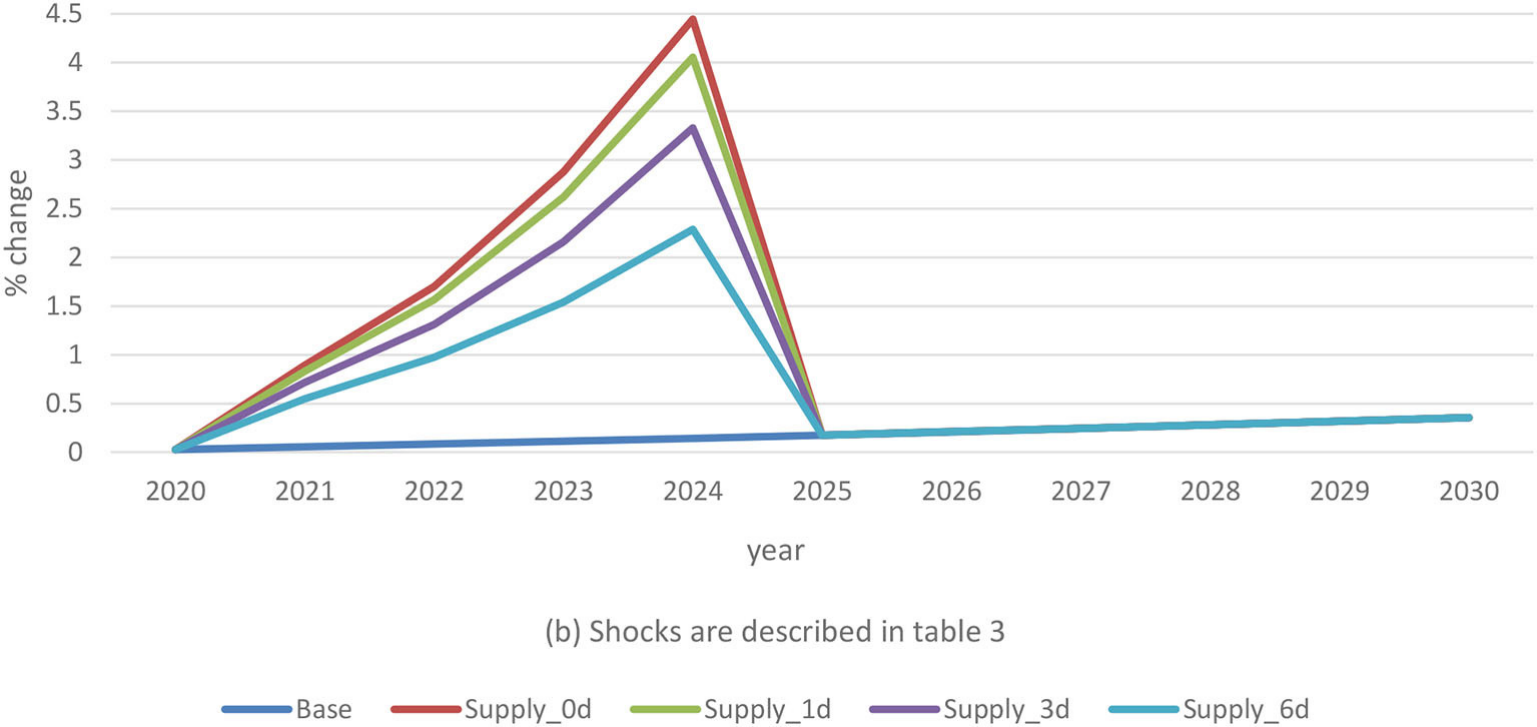
Commercial pig price changes in combined scenarios. (b) Shocks are described in Table 3.

The 15% supply shock acts on commercial pig prices by a dissimilar magnitude than the traditional sector. Prices ramp up by nearly 4.3% above baseline levels due to supply-only disruptions. Low levels of consumer avoidance reduce supply effects on price by only 0.3%, whereas the average and high demand shocks offset the supply-driven increased prices by 0.9% and 1.9%, respectively (Figure 5). The findings indicate significantly lower consequences of supply disruptions in the commercial sector than in the traditional sector. This accentuates the need for the development of effective ASF control and eradication policies that preserve smallholder producer livelihoods.

The traditional sector's combined supply shock with the 3% demand shock scenario is prolonged through 2030 to address different recovery schemes. Under an unrestricted recovery, ASF-related high prices converge almost immediately back to the baseline. The subsistence sector that constitutes most of the Haitian pig industry and the low level of biosecurity would complicate the implementation of a strategy that more likely guarantees prompt recovery from ASF. The appropriate policies are often achievable in a more extensive modern pig sector. Unlike the traditional sector, the high levels of biosecurity and technology growth in modern pig production enhance resilience to ASF supply and demand shocks.

**Figure 6:**
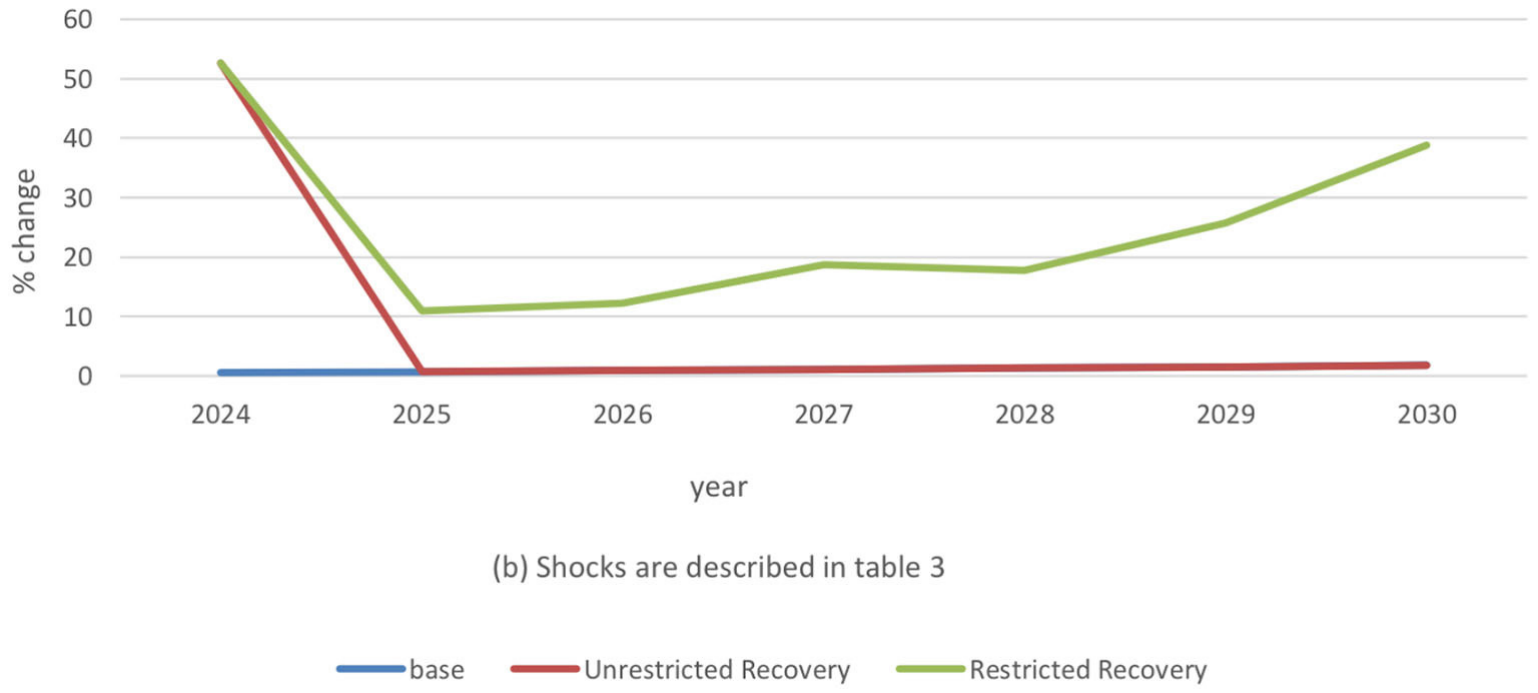
Traditional pig price changes in restricted recovery scenario. (b) Shocks are described in Table 3.

The restricted recovery scenario exemplifies Haiti's previous post-ASF experience. Under this scenario, an average of 7% decrease in ASF supply shock from 2024 to 2025 contributes to lower traditional pig prices by nearly 42% (Figure 6). Despite a continued, but narrower, supply shock reduction in the following years, prices tend to move in the opposite direction. Between 2025 and 2030, supply shocks drop by an additional 2%. Simultaneously, we noted a 28% price increase.

### Implications of swine price changes for producer income

The result discussion, in the previous section, centers around the price effects of ASF supply and demand-related shocks. However, price changes do not fully capture the losses to producers whose herds were infected. HPM-2021 does not include welfare measures. The estimated producer income and consumer spending capture elements of overall sector impacts. Producers' undiversified source of income, particularly in Haitian rural communities, amplifies the degree to which smallholder livelihoods may be affected by ASF shocks. Supplementary Table 5 summarizes the simulation results of ASF impacts on traditional pig producer income. In the baseline, the constant increase in pig production and prices contributes to higher income for both the traditional and the commercial pig sector until 2030. As observed in the price results, traditional producer average income growth by 2030 (6.01%) is larger than commercial producer income growth (0.81%), owing to a smaller commercial sector's share of total pork supply (Supplementary Tables 5, 6). Supplementary Tables 5, 6 are inserted in Supplementary material document.

The adverse impacts of ASF on domestic pork supply generate rapid price inflation until eradication of the disease. Lessons from previous outbreaks in Haiti have shown that many of the farmers that suffered from ASF losses received no compensation. Without a breeding stock and income source, many of those farmers fled the rural area in quest of new livelihoods. HPM-2021 simulation results indicate that in the short-term (2021-2024), the high prices generated by shortfalls in pork production are an opportunity to accelerate the income rise for the remaining farmers. This is likely an incentive to practice good biosecurity. However, as seen previously, high prices may have large consequences for food security and livelihood sustainability.

Under the stochastic (mean:15%) supply-only shock, remaining traditional producers would see their income increase by a minimum of 49% compared to the 2019 base year (Supplementary Table 5). Commercial producers, on the other hand, would benefit from an income rise ranging from 2.22% to 66% in 2024 compared to the 2019 base year (Supplementary Table 6). The unrestricted redevelopment of the swine sectors with new imported breeds, shows a noteworthy deceleration in income change. From 2025 to 2030, the commercial pig sector continued to experience an income growth similar to the baseline under the supply with no demand shock. The traditional sector's income growth, in contrast, is lower than the baseline growth. The scope of producer gains is conditioned by the extent of the supply shocks, the possibility to import new breeds to repopulate the industry, and consumers' avoidance of pork products.

Demand shocks resulting from consumer avoidance of pork products influence lower prices and have the opposite effect on swine producer income to a supply-only shock. A 3% demand shock with no supply disruptions would cause up to 0.26% of income losses to the traditional pig producers and 0.17% to commercial pig producers compared to the 2019 base year (Supplementary Tables 5, 6). In the combined scenarios, demand shocks act on income by offsetting a portion of the supply-induced income gain. The results indicate that, in both the traditional and commercial sectors, producer income tends to substantially lessen as consumers avoid pork products. Slower income growth is observed in the long term from both sectors, with the traditional pig sector showing a lower growth in the three combined scenarios than the baseline from 2027 to 2030.

As the swine industry recovers, the immediate slowdown of farmer income growth serves as a warning to traditional and commercial pig producers that the opportunities for gains following ASF infection are temporary. The market adjustment following ASF eradication through larger imports volumes from other countries and persisting consumer avoidance of pork products may create conditions for lower gains than the baseline forecasts in the long run. The model simulation does not account for consumers shifting to substitutable meats which would also limit the remaining pig producers' income growth during ASF. The country could also bring in greater pork imports *via* food security programs to lessen the price shocks to consumers.

### Implications of swine price changes for consumer expenditures

The demand shocks incorporated in HPM-2021 scenarios demonstrated the possibility of pork consumers to avoid pork following ASF detection. In many countries, pork avoidance may vary from 1 to 6% during the outbreak years, leading to substantial losses throughout the production system. In addition to changes in pork-consumption attitudes, ASF- induced pork price fluctuations and lower availability will influence consumer spending. Supplementary Tables 7, 8 summarize pork consumer spending trends with no disease shock until 2030 in comparison with various ASF outbreak scenarios. Supplementary Tables 7, 8 are inserted in Supplementary material document.

Supplementary Tables 7, 8 baseline results indicate a year-to-year increase in pork consumer expenditures in both pig sectors until 2030. This growth is particularly driven by a growing Haitian population (2.48% yearly). Despite a falling per capita income, higher expenditures are projected with the market incurring ASF supply shocks without any accounted consumer avoidance. In this scenario, traditional pork consumer expenditure rises by an average of 201% in 2024 compared to the 2019 base year (Supplementary Table 7). Commercial consumer expenditure, on the other hand, increases by only 3.11% in 2024 from the 2019 base year (Supplementary Table 8). Changes in the supply-only scenario are contrasted with the baseline changes from the same year to estimate consumers' change in well-being as a result of ASF shocks. By the last outbreak year (2024), traditional consumer losses could amount by an average of 200% from the baseline to the supply-only scenario, whereas commercial consumers would incur 2.89% percent in average losses.

Consumer avoidance of pork products contributes to lower losses in the traditional and commercial pig sector. A 1, 3, and 6% demand reduction would respectively offset those losses by 26, 60, and 87% in the traditional sector, and by 7, 32, and 64% in the commercial sector. The unrestrictive recovery from ASF would allow consumer expenditures to converge to baseline levels in 2025 in all scenarios. The incorporation of competitive sectors to pork in the model would alleviate expenditure losses as many consumers would shift toward other meats such as goat, chicken, or beef to avoid the high pork prices generated by the ASF outbreaks.

## Discussion

Pigs are one of the most plentiful animals on Haitian farms and are mostly owned by small farmers in rural areas. With minimal hard currency in rural communities, small-scale pig production serves as a source of wealth accumulation as well as a source of protein. Revenues from the sale of pigs comprise 30% of rural farm income, and are often used to finance children's education, weddings, funerals, and day-to-day activities. Being a high protein food and low risk investment, pigs play a role in diminishing food insecurity and social inequality in Haitian localities. The 2021 re-introduction of ASF is a major threat to the swine sector inventory still struggling to completely recover from the 1985 eradication. As evidenced by the past outbreaks, the undiversified income of traditional pig producers raises the risk of impoverishment from ASF high mortality and control measures.

HPM-2021 consists of a baseline (no-ASF) which indicates an expanding pig industry. Both pork producer income and consumer expenditure show a rising trend while the sector remains very self-sufficient, with minimal international trade involved. The first alternative scenario examined an ASF outbreak that is very small, such that no true change in production is experienced, but in which consumers avoid pork products. In this demand-only scenario, results suggest large income losses for traditional pig producers, driven by lower prices due to consumer avoidance of pork products. The smaller commercial sector experiences a lower scale of losses.

On the other hand, the 2021 ASF outbreak imposes adverse impacts on pork supply and consumer pork expenditures. With similar supply shocks implemented to both traditional and commercial pigs, results indicate disproportionately higher price consequences to the traditional pig sector. From the remaining pig producers' standpoint, the inflated prices show a possibility to earn additional income in the short term. However, as ASF is eradicated and the recovery process launched, the supply shock reduction associated with persisting consumer avoidance may contribute to a loss in long-term well-being. Consumers, on the other hand, will undergo increased pork expenditures during the ASF outbreak period, resulting in lower well-being until 2030. The lack of a welfare measure for impacted swine owners is a limitation of this analysis and the subject of future research.

The inflated prices resulting from ASF are a temporary windfall for remaining pig producers able to take advantage of fewer competitors during the outbreak period. Although partially offset by consumer avoidance of pork, high prices leading to higher incomes for non-infected farms may incentivize traditional pig producers to not report ASF symptoms on their farms. Past studies have indicated that high market prices and high compensation can create perverse incentives for reporting (41). The willingness to report disease will vary with government responses and efficient livestock valuation to determine compensation rates (42). On the other hand, if it becomes public knowledge that producers are not reporting disease, those actions may trigger a loss of consumer confidence which can almost equally counteract the supply effects on price. Besides the overall impacts on production, the high prices of pork products may stimulate consumers' shift to cheaper imports or alternative protein sources. This was not estimated by HPM-2021 and is a subject for future research.

There are no recent assessments of ASF implications in the Caribbean, and few models that evaluate the economic impacts of animal health response policies in less developed countries like Haiti. The lack of price data is one of the constant challenges to economic impact analysis in Haiti. Commodity prices are not regularly collected. As a result, HPM-2021 uses a combination of historical price gaps with the Dominican Republic and inflation adjustments to estimate year-to-year Haitian prices until 2019. In addition to price, no set of elasticities is readily available for Haiti. HPM-2021 elasticities are calculated based on previous years data as described in the data section.

This study offers a starting point for additional work on animal health response in the Caribbean. A more developed assessment of the economic impacts of ASF in Haiti will include welfare measurements from supply and demand relationships. The welfare analysis will capture the changes in swine and maize markets' producer and consumer surpluses, as well as the multimarket “domino” effects that may be originated from distortions in the swine sector. Future modeling activities may also be conducted to assess the unrestricted ASF impacts on other related sectors of Haiti's economy, such as crop inputs and substitutable meats like chicken and goat. Including additional swine feeds and competitive meats would broaden the producer costs calculation and consumer shocks due to the opportunity to shift consumption to other non-infected products. This would involve cross-sectoral data gathering and elasticity calculations to influence variations in pork prices and well-being. Lastly, the analysis may include a vaccine development scenario. A vaccination scenario would require experimental data about the evolving ASF vaccine, or the historical effectiveness of vaccines in reducing the impacts of related diseases, such as Classical Swine Fever.

High prices could hinder development initiatives in the mostly poor rural communities and accentuate the lack of access to food, health, education, and other essential services. The unrestricted repopulation strategy results in the rapid recovery of swine inventories. Alternatively, when repopulation is restricted to reflect historic barriers faced during the 1984 PEPPADEP recovery, longer term losses occur as a result of ASF in 2021. The results imply many socioeconomic consequences could occur. The prospective impacts on educational attainment, food security, and migration outline the need to develop effective pro-poor ASF eradication and repopulation strategies.

## Data availability statement

The original contributions presented in the study are included in the article/Supplementary material, further inquiries can be directed to the corresponding author.

## Author contributions

KR designed the original model for the study. RJ-P readapted and contextualize the model to the Haitian livestock system. AH and RJ-P conceived the scenarios and conducted the results interpretation. All authors made direct intellectual contribution to the article and approved it for publication. All authors contributed to the article and approved the submitted version.

## Funding

RJ-P's time was partially funded under grant funding from the United States Department of Agriculture (USDA) Animal and Plant Health Inspection Service (APHIS) Veterinary Services (VS) National Animal Health Preparedness and Response Program (NADPRP) under agreement number AP20VSCEAH00C031. All analyses and conclusions are those of the authors and do not represent the positions or policies of USDA-APHIS-VS.

## Conflict of interest

The authors declare that the research was conducted in the absence of any commercial or financial relationships that could be construed as a potential conflict of interest.

## Publisher's note

All claims expressed in this article are solely those of the authors and do not necessarily represent those of their affiliated organizations, or those of the publisher, the editors and the reviewers. Any product that may be evaluated in this article, or claim that may be made by its manufacturer, is not guaranteed or endorsed by the publisher.

## Abbreviations

ASF: African Swine Fever
FAO: Food and Agriculture Organization of The United Nations
USAID: United States Agency for International Development
USDA: United States Department of Agriculture.
