# Supplementary material for: An analysis of African Swine Fever consequences on rural economies and smallholder swine producers in Haiti
Source: Front Vet Sci. 2022 Oct 12;9:960344. doi: 10.3389/fvets.2022.960344 (PMC9597192; doi:10.3389/fvets.2022.960344)
Supplement: Supplementary file 1 [file Data_Sheet_1.docx]

Supplementary Material

Three sections:

Section 1: Core Equations Section 2: Supplementary Result Tables Section 3: Dataset

# Core Equations

1. Domestic maize supply functions

Maize supply is a function of area (equation 1) and yield (equation 2). Maize will subsequently be used for both human consumption and animal consumption. For the purposes of this study, the sum of human consumption of domestic maize and swine consumption of domestic maize will equal total domestic maize production; however, this assumption is controlled in the structure of the feed and food equations described in the following sections (equations 7 and 9). Maize demand for both feed and food can also include imported maize. These are defined in (11) and (12).

$\log\left( A_{rt} \right)=$ $\alpha_{rt}+ \varepsilon_{1_{r}}^{maize}\times\log\left( P_{rt}^{maize} \right)+\sum_{l} (\varepsilon_{1_{lr}}^{swine}\times log(P_{lrt}^{swine}))$ (1)

$\log\left( Y_{rt} \right)=$ $\gamma_{rt}+ \varepsilon_{2_{r}}^{maize} \times\log\left( P_{rt}^{maize} \right)+\sum_{l} (\varepsilon_{2_{lr}}^{swine}\times log(P_{lrt}^{swine}))$ (2)

In equations (1) and (2), $A\mathrm{and}Y$ are respectively the planted area and the yield of maize in each production region r$\in${Haiti, Dominican Republic} in each year that is modeled t$\in${2019,…, 2030}. $P^{maize}$ is the producer price of maize, and $P^{swine}$ is the producer price of each livestock sector $l\in${traditional pig, commercial pig} at any given year. The intercept of maize area function is notated $\alpha$. The maize planted area equation (1) also consists of $\varepsilon_{1}^{maize}$, the elasticity of maize area to maize prices and $\varepsilon_{1}^{swine}$, the elasticity of maize area to prices for each type of livestock in each region and any given year. The maize yield intercept ($\gamma$) is explained further in equation 4. The elasticity of maize yield with respect to maize prices ($\varepsilon_{2}^{maize}$), and the elasticity of maize yield to price of each type of livestock ($\varepsilon_{2}^{swine}$) are included in the maize yield equation. Elasticities are held constant across time (t).

The area and yield equations are combined to define the maize supply available at any given year. Maize makes up a key portion of swine feeds in Haiti for both traditional and commercial pig production. A change in maize production and price will affect the pork industry and vice-versa. The maize supply equation establishes the relationship between the quantity of maize produced and changes in the production factors and demand by the swine sectors.

$\alpha_{rt}=\log\left( A_{0_{r}} \right)- \varepsilon_{1_{r}}^{maize}\times log(P_{0_{rt}}^{maize})-\sum_{l} (\varepsilon_{1_{lr}}^{swine}\times log(P_{0_{lrt}}^{swine}))$ (3)

$\gamma_{R,T}=\log\left( Y_{0_{r}} \right)-\varepsilon_{2_{r}}^{maize} \times\log\left( P_{0_{rt}}^{maize} \right)-\sum_{l} {(\varepsilon}_{2_{lr}}^{swine}\times\log\left( P_{0_{lrt}}^{swine}) \right)$ (4)

Equation 3 formulates the intercept of maize area $(\alpha)$ function for each region. It includes$A_{0}$, which is the original (2019) maize area in hectare for consumption in each region, $\varepsilon_{1}^{maize}$ and $\varepsilon_{1}^{swine}$, which are defined in equation 1. Included in both equations 3 and 4, $P_{0}^{maize}$ is the baseline producer price of maize, and $P_{0}^{swine}$ is the baseline producer price of each livestock sector ($l$) in Dominican Pesos. The maize yield intercept ($\gamma$), on the other hand, encompasses the 2019 maize yield in tonne per hectare ($Y_{0})$ for each region, $\varepsilon_{2}^{maize}$, and $\varepsilon_{1}^{swine}$, also defined in equation 2.

1. Livestock supply functions

Livestock supply (equation 5) in the model is limited to swine production and consists of $l\in${traditional pigs, commercial pigs}. Traditional pig production makes up the majority of swine farms and live pig inventory in both Haiti and the Dominican Republic (r). In both swine sectors the main feed grain is maize, primarily produced domestically.

$\log(S_{lrt})=$ $\delta_{lrt}+ \sum_{l} {(\varepsilon}_{3_{lr}}^{swine}\times log(P_{lrt}^{swine}))+{(\varepsilon}_{3_{lr}}^{maize} \times\log\left( P_{rt}^{maize} \right)$ (5)

$\delta_{lrt}=\log\left( {S_{0}}_{lr} \right)- \sum_{l} {(\varepsilon}_{3_{lr}}^{swine}\times log(P_{0_{lrt}}^{swine}))-\varepsilon_{3_{lr}}^{maize} \times\log\left( P_{0_{rt}}^{maize} \right)$ (6)

The livestock supply equation follows a very similar procedure to the maize supply equation. It includes a double-log intercept of livestock supply ($\delta)$ stated in equation 6. The intercept of livestock supply ($\delta$) is a function of $S_{0}$; the 2019 traditional and commercial pigs ($l$) produced in each region (r); the elasticity of livestock supply with respect to own and cross swine output prices, $\varepsilon_{3}^{swine}$; and the elasticity of livestock supply to the price of maize, $\varepsilon_{3}^{maize}$. $P_{0}^{maize}$is the baseline producer price of maize, and $P_{0}^{swine}$ is the baseline swine producer price in each region.

In equation (5), $S$ is the supply of livestock at any given time. Equation 5 is made up of the intercept and elasticities ($\delta, \varepsilon_{3}^{swine}$, $\varepsilon_{3}^{maize}$). $P^{maize}$ is the producer price of maize, and $P^{swine}$ is the producer price of livestock.

1. Food demand functions

$\log(D_{frt})=\vartheta_{frt}+ \sum_{f} \left( \varepsilon_{fr}^{food}\times\log\left( Z_{frt} \right) \right)+\varepsilon_{fr}^{income}\times\log\left( I_{rt} \right)$ + $\log\left( H_{rt} \right)$ (7)

$\vartheta_{frt}=\log\left( D_{0_{fr}} \right)-\sum_{f} \left( \varepsilon_{fr}^{food}\times\log\left( {Z_{0}}_{frt} \right) \right) -\varepsilon_{fr}^{income}\times\log\left( I_{rt} \right)-log(H_{rt})$ (8)

Both pork and maize are largely used for human consumption in Haiti and the Dominican Republic. The double-log food demand equation (7) defines the potential impacts of maize price, livestock price, and consumer income alterations on the quantity of food consumed $f\in${food maize, traditional pork, commercial pork}. The food demand function ($D$) consists of $\varepsilon^{food}$, the elasticity of food demand with respect to own and cross food prices, $\varepsilon^{income}$, the elasticity of food demand to consumer income in each region ($r)$and $Z$, the consumer price of maize and swine products at any given time ($t)$. $H$, which is the total human population at time ($t$), adjusts food demand changes as a result of year-to-year fluctuations in population. $\vartheta$, formulated in (8) is also included.

The intercept of food demand function ($\vartheta$) is dependent of $D_{0}$, the 2019 food demand in Haiti and the Dominican Republic ($r)$, the previously described elasticities $\varepsilon^{food}$ and $\varepsilon^{income}$, as well as $Z_{0}$, the baseline consumer price of maize and swine products. Included in both (7) and (8), $I$ is the income per capita in time $(t)$ for Haiti and the Dominican Republic.

1. Feed demand functions

$\log\left( K_{crt} \right)=\varphi_{crt}+\log\left( \sum_{l} \left( S_{lrt} \right) \right)+\varepsilon_{4_{cr}}^{maize}\times\log\left( Z_{crt}^{maize} \right)$

$+ \sum_{l} \left( \varepsilon_{4_{lr}}^{swine}\times\log\left( P_{lrt}^{swine} \right) \right)$ (9)

Maize domestically produced in Haiti and the Dominican Republic is often used to feed traditional and commercial pigs, $c\in${feed maize}. In equation (9), $K,$which is the maize demand for swine feeding purpose at a given year ($t$) is a function of livestock supply $S$, defined in equation (5). $\varepsilon_{4}^{maize}$ is the maize demand elasticity with respect to maize consumer price ($Z^{maize})$ at time $t$, and $\varepsilon_{4}^{swine}$ is the elasticity of maize demand to traditional and commercial pig producer prices ($P^{swine}$). $\varphi$, the intercept of maize demand is also included.

$\varphi_{crt}=\log\left( K_{0_{cr}} \right)-\log\left( \sum_{l} \left( S_{0_{lr}} \right) \right)- \varepsilon_{4_{cr}}^{maize}\times\log\left( Z_{0_{crt}}^{maize} \right)$

$-\sum_{l} \left( \varepsilon_{4_{lr}}^{swine}\times\log\left( P_{0_{lrt}}^{swine} \right) \right)$ (10)

Equation (10) formulates the intercept of maize demand ($\varphi)$ for each pig sector of each region in the model. It consists of the coefficient $\varepsilon_{4}^{maize}$, elasticity of maize demand with respect to own baseline consumer price ($Z_{0}^{maize})$, and $\varepsilon_{4}^{swine}$, the elasticity of maize demand to producer prices ($P_{0}^{swine}$) of each livestock sector. $S_{0}$is the 2019 livestock supply and $K_{0}$, the 2019 feed demand by both livestock sectors.

1. Balance Equations

HPM-2021 has no objective function included (Rutherford, 1995). A set of inequalities are formulated to exploit the complementarity links between various equations. The model includes a product inflow equation that ensures, under regular market conditions (no disease shocks), that domestic supply and imports of feed and food meet or surpass local demand (11). Outflow inequalities are also constructed to formulate that all produced maize ($j$)—consisting of both previously described feed maize ($c$) and food maize — and livestock supply is equal or greater than local demand and export as shown in equation (12).

$\sum_{rr} (Q_{jlrrt}+M_{jlrrt})\geq$ $D_{flrt}$ + $N_{jr}\times K_{crt}$ (11)

Equation (11) is the inflow inequality where, $Q$ is the transport of goods from one region to another $rr\in${Haiti-Dominican Republic-rest of the Caribbean-rest of the world}. $M$ is the import of swine products and maize in tonnes from the rest of the Caribbean and the rest of the world. $D$and K, also included, are respectively defined in equation (7) and equation (9). $N$ is the identity matrix for proper dimension of total feed maize and food maize demand in inflow.

$\sum_{rr} {(Q}_{jlrrt}$ + $X_{jlrrt})\leq\sum_{l} {(O}_{lr}\times S_{lrt})$ + $\sum_{j} {(N}_{jr}\times V_{jrt})$ (12)

Equation 12 is the outflow inequality which consists of X, is the total maize and swine products ($l)$ exported at time $t$from Haiti and the Dominican Republic to the rest of the Caribbean and the rest of the world ($rr$), and $O$, the identity matrix for proper dimension of livestock supply in outflow. $S$ is the supply of livestock and $V$ is the total supply of both feed and food maize at time t.

# Supplementary Tables

**Table 5.** Summary Statistics of ASF Impacts on Haitian Traditional Pig Producers' Change in Income from the 2019 Base Year**^[[1]](#footnote-1)^**

**Table 6**. Summary Statistics of ASF Impacts on Haitian Commercial Pig Producers' Change in Income from the 2019 Base Year

**Table 7**. Summary Statistics of ASF Impacts on Haitian Traditional Pig Consumers' Change in expenditures from the 2019 Base Year**^[[2]](#footnote-2)^**

**Table 8**. Summary Statistics of ASF Impacts on Haitian Commercial Pig Consumers' Change in expenditures from the 2019 Base Year

## 3 Dataset

**A.1.** Market Parameters

|  | CONV | PMARGPR | CTA | XPRO | FEEDCONV |  |
| --- | --- | --- | --- | --- | --- | --- |
| MAIZE | 0.90 | 0.10 | 1 |  |  |  |
| TRADPIG | 0.50 | 0.05 | 3 |  | 3.8 |  |
| COMMPIG | 0.50 | 0.05 | 3 | 10000 | 3.0 |  |

“CONV” is the conversion ratio from consumer weight to producer weight, “PMARGPR” the processing costs, “CTA” the cost of transportation for each commodity, “XPRO” the extra processing costs associated with exports, and “FEEDCONV is the feed-meat conversion ratio.

**A.2.** Original crop area for urban-rural consumption 2019 (1000ha)

|  |  | URBAN | RURAL | **TOTAL** |  |  |
| --- | --- | --- | --- | --- | --- | --- |
| MAIZE | HT | 221.860 | 77.72 | **299.58** |  |  |
| MAIZE | DR | 17.209 | 12.058 | **29.27** |  |  |
| MAIZE | CB | 286.028 | 174.124 | **460.15** |  |  |

Source: FAOSTAT data.

**A.3.** Original crop yield for urban-rural consumers 2019 (ton per har)

|  |  | URBAN | RURAL | **TOTAL** |  |  |
| --- | --- | --- | --- | --- | --- | --- |
| MAIZE | HT | 0.80 | 0.80 | **0.80** |  |  |
| MAIZE | DR | 1.87 | 1.87 | **1.87** |  |  |
| MAIZE | CB | 1.40 | 1.40 | **1.40** |  |  |

Source: Authors’ estimates based on FAOSTAT data.

**A.4.** Urban & rural per capita income in 2019 (DR Pesos per person per yr)

|  | URBAN | RURAL | TOTAL |  |
| --- | --- | --- | --- | --- |
| HT | 702.760 | 568.561 | 642.152 |  |
| DR | 6708.515 | 1573.602 | 5736.692 |  |
| CB | 7352.340 | 3003.068 | 6105.091 |  |
|  | 6350.834 | 2239.128 | 5126.054 |  |

Source: WorldBank Database

| **A.5.** Annual income growth 2019-2020 | | | |
| --- | --- | --- | --- |
|  | URBAN | RURAL | TOTAL |
| HT | -0.035 | -0.025 | -0.0305 |
| DR | -0.080 | -0.040 | -0.0783 |
| CB | -0.080 | -0.030 | -0.0728 |

**A.6.** Original production oriented for urban & rural consumer groups 2019 (1000tons)

|  |  | URBAN | RURAL | **TOTAL** |  |  |  |
| --- | --- | --- | --- | --- | --- | --- | --- |
| MAIZE | HT | 177.488 | 62.179 | **220** |  |  |  |
| MAIZE | DR | 32.181 | 22.548 | **49.840** |  |  |  |
| MAIZE | CB | 400.439 | 243.774 | **586.330** |  |  |  |
| TRADPIG | HT | 11.970 | 10.441 | **22.411** |  |  |  |
| TRADPIG | DR | 13.202 | 8.382 | **21.584** |  |  |  |
| TRADPIG | CB | 118.791 | 66.754 | **185.545** |  |  |  |
| COMMPIG | HT | 6.580 | 3.024 | **9.604** |  |  |  |
| COMMPIG | DR | 24.763 | 13.609 | **38.372** |  |  |  |
| COMMPIG | CB | 118.791 | 66.754 | **185.545** |  |  |  |

Source: FAOSTAT data.

The distribution of commodity production for urban and rural consumer is proportional to the urban and rural consumption

| **A.7**. [Original consumer price 2019 (DR Pesos per kg)](file:///C:\Users\rjeanpi\Documents\Sensitivity%20Analysis%20HPM2021\HPM_Input.xlsx#RANGE!A1) | | | | |
| --- | --- | --- | --- | --- |
|  |  | URBAN | RURAL | **TOTAL** |
| MAIZE | HT | 612.20 | 612.20 | **612.20** |
| MAIZE | RD | 498.88 | 498.88 | **498.88** |
| MAIZE | CB | 993.10 | 993.10 | **993.10** |
| TRADPIG | HT | 4870 | 3030 | **3950.00** |
| TRADPIG | DR | 1979 | 1979 | **1978.88** |
| TRADPIG | CB | 4889 | 4889 | **4888.90** |
| COMMPIG | HT | 4870 | 4030 | **4450.00** |
| COMMPIG | DR | 1979 | 1979 | **1978.88** |
| COMMPIG | CB | 4889 | 2390 | **3639.45** |

Source: Prices are gathered from the USAID 1983 “Interim Swine Repopulation” and FAOSTAT. 2019 Haitian Prices are calculated based on historical gap with Dominican prices, accounting inflation.

**A.8.** Original per capita food cons. 2019 (kg per per year)

|  |  | URBAN | RURAL | **TOTAL** |  |
| --- | --- | --- | --- | --- | --- |
| MAIZE | HT | 28.054 | 18.286 | **23.17** |  |
| MAIZE | DR | 89.978 | 367.950 | **228.96** |  |
| MAIZE | CB | 58.886 | 123.999 | **91.44** |  |
| TRADPIG | HT | 1.298 | 1.400 | **1.35** |  |
| TRADPIG | DR | 2.374 | 6.457 | **4.42** |  |
| TRADPIG | CB | 2.715 | 3.794 | **3.25** |  |
| COMMPIG | HT | 0.951 | 0.540 | **0.75** |  |
| COMMPIG | DR | 3.119 | 7.342 | **5.23** |  |
| COMMPIG | CB | 2.715 | 3.794 | **3.25** |  |

Source: Ralph’s estimates based on FAOSTAT data. The per capita food consumption is estimated based on production, trade, and commodity conversion ratios from producers to consumers wight gathered from FAOSTAT.

| **A.9.** Urban & rural population in 2019 (1000 inhabitants) | | | | | | | | | |
| --- | --- | --- | --- | --- | --- | --- | --- | --- | --- |
|  | URBAN | | RURAL | | **TOTAL** | |  | | |
| HT | 6143.008 | | 4969.937 | | **11123.18** | |  | | |
| DR | 8823.303 | | 2059.693 | | **10883** | |  | | |
| CB | 31492.82 | | 12662.505 | | **44155.32** | |  | | |
|  | **46459.13** | | **19692.135** | | **66161.5** | |  | | |
| **A.10.** Annual population growth 2019-20 (fraction) | | | | | | | | |  |
|  | | URBAN | | RURAL | | TOTAL | |  |  |
| HT | | 0.0301 | | 0.0183 | | 0.0248 | |  |  |
| DR | | 0.0397 | | 0.0456 | | 0.0408 | |  |  |
| CB | | 0.0216 | | 0.0321 | | 0.0247 | |  |  |

Source: FAOSTAT and WorldBank Database

| **A.11.** Elasticities  Elasticity of crop area with respect to output price | | | | | | | | |  |  |  |
| --- | --- | --- | --- | --- | --- | --- | --- | --- | --- | --- | --- |
|  | | | HT | DR | | CB | | |  |  |  |
| MAIZE | | | 0.37 | 0.36 | | 0.34 | | |  |  |  |
| Elasticity of crop yield with respect to output price | | | | | | | | | |  |  |
|  | | | HT | DR | | CB | | | |  |  |
| MAIZE | | | 0.01 | 0.41 | | 0.22 | | | |  |  |
| Elasticity of livestock supply with respect to output prices | | | | | | | | | | |  |
|  |  | | | | HT | | DR | CB | | |  |
| TRADPIG | TRADPIG | | | | 0.900 | | 0.610 | **0.900** | | |  |
| TRADPIG | COMMPIG | | | | -0.620 | | -0.030 | **-0.620** | | |  |
| COMMPIG | TRADPIG | | | | -0.620 | | -0.030 | **-0.620** | | |  |
| COMMPIG | COMMPIG | | | | 0.900 | | 0.610 | **0.900** | | |  |
| [Elasticity of livestock supply with respect to feed prices](file:///C:\Users\rjeanpi\Documents\Sensitivity%20Analysis%20HPM2021\HPM_Input.xlsx#RANGE!A1) | | | | | | | | | | | |
|  | | MAIZE | | | | | | | | | |
| TRADPIG | | -0.002 | | | | | | | | | |
| COMMPIG | | -0.002 | | | | | | | | | |

[Food demand elasticity with respect to consumer income](file:///C:\Users\rjeanpi\Documents\Sensitivity%20Analysis%20HPM2021\HPM_Input.xlsx#RANGE!A1)

|  |  | URBAN | RURAL | **TOTAL** |
| --- | --- | --- | --- | --- |
| MAIZE | HT | 0.467 | -1.417 | **-0.113** |
| TRADPIG | HT | 0.986 | 1.336 | **0.014** |
| COMMPIG | HT | 1.095 | 1.470 | **0.065** |

[Food demand elasticities with respect to food prices](file:///C:\Users\rjeanpi\Documents\Sensitivity%20Analysis%20HPM2021\HPM_Input.xlsx#RANGE!A1)

|  |  |  | **TOTAL** |
| --- | --- | --- | --- |
| MAIZE | MAIZE | HT | **-0.461** |
| MAIZE | TRADPIG | HT | **0.058** |
| MAIZE | COMMPIG | HT | **0.058** |
| TRADPIG | MAIZE | HT | **-0.002** |
| TRADPIG | TRADPIG | HT | **-0.013** |
| TRADPIG | COMMPIG | HT | **0.339** |
| COMMPIG | MAIZE | HT | **-0.241** |
| COMMPIG | TRADPIG | HT | **0.083** |
| COMMPIG | COMMPIG | HT | **-0.384** |

1. Each of the shock categories and characteristics are described in table 3 [↑](#footnote-ref-1)
2. Each of the shock categories and characteristics are described in table 3 [↑](#footnote-ref-2)
